# Impact of diabetes on gastrointestinal and urinary toxicity after radiotherapy for gynecologic malignancy

**DOI:** 10.4274/tjod.galenos.2019.56957

**Published:** 2020-02-28

**Authors:** Emine Elif Özkan, Evrim Erdemoğlu, Jalal Raoufi

**Affiliations:** 1 Süleyman Demirel University Faculty of Medicine, Department of Radiation Oncology, Isparta, Turkey; 2 Süleyman Demirel University Faculty of Medicine, Department of Gynecologic Oncology, Isparta, Turkey

**Keywords:** Diabetes, gynecologic tumor, radiation toxicity, pelvic radiotherapy

## Abstract

**Objective:**

Although diabetes is a common co-morbidity in patients with gynecologic cancer, information about its impact on radiation toxicity in patients with gynecologic cancer treated with external pelvic irradiation is scarce. We aimed to investigate the relation of diabetes with acute toxicity in patients with gynecologic tumors who underwent pelvic +/- paraaortic radiotherapy.

**Materials and Methods:**

One hundred twenty-nine patients with endometrium or cervix carcinoma were enrolled in the study. Demographic features, presence of diabetes, incidence and severity of upper gastrointestinal (UGIS), lower gastrointestinal (LGIS), and urinary symptoms were recorded from files. Correlation and logistic regression analysis was used to determine the impact of diabetes, age, chemotherapy, paraaortic irradiation on toxicities, and a prediction model was developed.

**Results:**

The median age of 77 patients with endometrium cancer and 52 cervix cancer was 61 (range, 25-92) years, and 28 (21.7%) of them had diabetes. The median pelvic and tumor/tumor bed dose was 5040+247.65 cGy and 5040+222.91 cGy, respectively. Age and Gr 0 UGIS toxicity were significantly related (p=0.047). LGIS Gr 0 toxicity was found to be significantly higher in patients with diabetes (p=0.045). Gr 0 and 2 UGIS toxicities were both found to be significantly correlated with paraaortic irradiation (both p<0.001). Diabetes is also an important determinant on UGIS toxicity in patients who underwent paraaortic irradiation.

**Conclusion:**

The correlation we found between toxicity and diabetes, concurrent chemotherapy or paraaortic radiation necessitates special care and risk stratification for patients with diabetes. Further prospective studies with long follow-up and larger patient groups are warranted.

**PRECIS:** In 129 gynecologic tumor patients we investigated the impact of diabetes on radiation toxicity.

## Introduction

Diabetes is one of the common comorbidities in patients with cancer, leading to long-term complications^(1)^. The impact of diabetes mellitus on radiation toxicity of lung and rectum is reported by a number of previous studies. Normal lung tissue toxicity in terms of radiation pneumonitis is proved to be higher in diabetic patients with lung cancer^(2,3,4,5)^. Radiographic radiation-induced lung injury has also found to be associated with the presence of diabetes after lung stereotactic body radiation therapy, most prominently early after treatment. Increased caution while treating patients with diabetes is strongly suggested^(6)^. In patients with prostate cancer treated with pelvic radiotherapy, the association of a high incidence and high-grade incontinence and sexual function^(7)^, other genitourinary symptoms^(8)^ with diabetes has also been reported. Even in patients with localized prostate cancer, a negative effect of diabetes on late gastrointestinal and urinary toxicities has been found^(9)^. Kalakota et al.^(8)^ suggested taking the relationship into consideration in patients with diabetes, especially among those receiving dose-escalated RT or with a history of surgery. Even the newer techniques such as intensity modulated radiotherapy (IMRT) or image-guided radiotherapy, anatomic close proximity of rectum and lower urinary tract causes symptoms leading to impairment in quality of life^(10)^. The effect of diabetes on radiation toxicity has been the subject of debate in many studies with patients with prostate carcinoma. However, it has not been investigated in gynecologic tumors; therefore, we aimed to determine whether diabetes had any impact on the acute radiation adverse effects of women who underwent pelvic radiation therapy for gynecologic malignancies.

## Materials and Methods

The study was approved by the Scientific Research Ethics Committee of the medical faculty of Süleyman Demirel University (protocol code: 2019/139). All procedures were performed in terms of the ethical standards of the institutional research committee in alliance with the 1964 Helsinki Declaration and its later amendments. Informed consent was waived owing to the retrospective nature of the study.

The medical records and laboratory data of 129 patients with gynecologic tumors who underwent pelvic +/- paraaortic radiotherapy from September 2011 to January 2019 were evaluated retrospectively. The inclusion criteria were: (1) patients who were diagnosed and histologically confirmed as having endometrium or cervix carcinoma; (2) patients who underwent primary radical chemoradiotherapy or adjuvant radiotherapy; (3) patients who received a dose of radiotherapy ranging between 4500 cGy-5400 cGy in 25-30 fractions; and (4) patients who acquired 3D conformal radiotherapy (3DCRT) or IMRT. The exclusion criteria were: (1) patients with missing data in terms of toxicity recording; (2) patients who had known chronic symptomatic proctitis; (3) symptomatic hemorrhoids; (4) and those who had known previous urinary or rectal surgery.

Taking the above-mentioned criteria into consideration, 77 patients with endometrium carcinoma and 52 with cervix carcinoma were included in the study. Radiotherapy was given to the target volume delineated with the guidance of preoperative fluorodeoxyglucose-positron emission tomography/computed tomography  regarding high sensitivity, specificity, and negative predictive values for detecting pelvic and paraaortic lymph node metastasis as reported previously^(11)^.

The severity of radiation-induced upper gastrointestinal (UGIS) (nausea, vomiting, loss of appetite, weight loss), lower gastrointestinal (LGIS) (hemorrhoids, diarrhea, rectal bleeding) and urinary symptoms (dysuria, pollakuria, polyuria, hematuria, urgency) are graded and recorded according to the Radiation Oncology Toxicity Grading (RTOG) grading system (Table 1)^(12)^. Correlation and logistic regression analysis were used to determine the impact of diabetes status, age, concomitant chemotherapy, and paraaortic irradiation on the grades of toxicities. Additionally, a toxicity probability predictor model was developed.

## Results

The median age of entire cohort was 61 (range, 25-92) years. The clinical and demographic characteristics of the study participants are shown in Table 2. Radiotherapy was given with curative intent in 23 patients who were inoperable and 6 patients with cervix carcinoma who underwent paraaortic lymphatic sampling. The number of patients treated with 3DCRT, static IMRT, and IMRT were 86, 37, and 6, respectively. The median pelvic dose was 5040 (4500-6120) cGy, total dose to tumor or tumor bed was 5040 (4500-6120) cGy. Of the 129 patients, 28 had type II diabetes.

### Statistical Analysis

**Figure 1 a-b:**
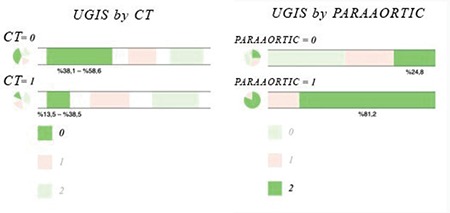
Upper gastrointestinal toxicity according to grades with concomitant chemotherapy (a) and paraaortic radiotherapy (b) UGIS: Upper gastrointestinal

**Figure 2 a-b:**
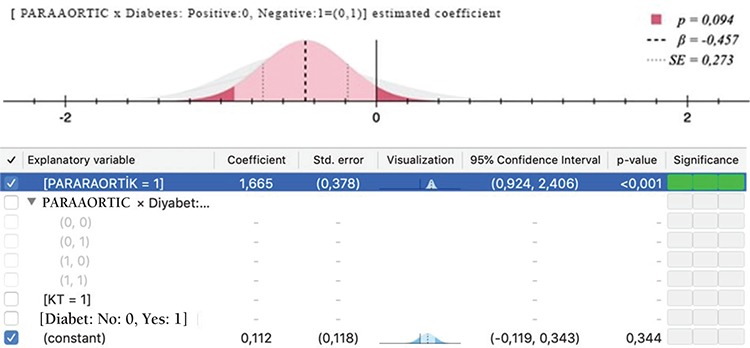
Standard error and confidence intervals for predictor effect of pararaortic irradiation

UGIS toxicity was significantly related with concomitant chemotherapy (Figure 1a) and paraaortic radiotherapy (Figure 1b) with p<0.001. The difference in UGIS toxicity according to the presence of diabetes was not significant. With Pearson correlation, age and Gr 0 UGIS toxicity was found significantly related (p=0.047). When paraaortic radiotherapy and UGIS toxicity correlation was evaluated using the z-score test, Gr 0 and 2 toxicities were both found to be significantly correlated (both p<0.001); however, the result was statistically nonsignificant for Gr 1 (p=0.383). Concomitant chemotherapy and UGIS toxicity was found to be statistically significantly correlated in the chi-square test (p=0.02). LGIS Gr 0 toxicity was found to be related with diabetes (p=0.045). Pearson’s test revealed a significant correlation between diabetes and LGIS (p=0.037). In addition, a significant correlation with concurrent chemotherapy was found (p=0.042). No significant correlation was found with diabetes and urinary toxicity in the z test. Concurrent chemotherapy and urinary toxicity correlation was evaluated using z scores, which revealed statistical significance for Gr 2 toxicity (p=0.012). UGIS, LGIS, and GUS toxicity ratios according to diabetes and correlation results are given in Table 3. The correlation results of chemotherapy and paraaortic radiotherapy with toxicity in relation with having diabetes are summarized in Table 4. An ordinal regression analysis was performed and variables with p<0.1, which were concurrent chemotherapy, paraaortic irradiation, diabetes, blood glucose level, and age, were included in the model. A toxicity prediction model for pelvic radiation treatment in this study was developed using a regression test. Paraaortic irradiation was significant in predicting LGIS (p=0.09) and UGIS toxicity (p<0.001). The standard error and confidence intervals are shown in Figure 2a and 2b. The prediction values are shown in Table 5. Diabetes was not found to be predictive for UGIS toxicity. No significant predictor was found for urinary toxicity in the regression analysis.

## Discussion

In this study, we evaluated the impact of diabetes on the incidence and severity of UGIS, LGIS, and urinary acute radiation toxicity in patients with gynecologic cancer who underwent pelvic +/- paraaortic radiotherapy. The relation of diabetes with radiation toxicity should not be unexpected if one considers previous reports indicating increased radiation-induced pulmonary toxicity after thoracic irradiation and proctitis or urinary incontinence consequent to pelvic radiotherapy with diabetes mellitus^(2,3,4,5,7)^. This connection has been enucleated with a number of theories. The increased morbidity and mortality rates in patients with diabetic cancer after surgery was reported previously and attributed to reduced leukocyte activities, such as phagocytosis and opsonization, which consequently intervene in natural host immunity^(13,14)^. Radiation causes more significant damage in fast proliferating tissues such as the lining epithelium of the skin, gastrointestinal tract, and blood vessels. The vascular configuration, which plays the major role in the repair of radiation damage, becomes disrupted through an activated coagulation system, decreased blood flow, thrombosis, and capillary necrosis^(14,15,16)^. Diabetes leads to an impairment in the function of vessel endothelial lining and dilatation of microvasculature^(14)^, resulting in dysfunctional tissue repair. Our results revealed that patients without diabetes mellitus had a significantly lower incidence of LGIS toxicity in patients with diabetes, which we proved with a significantly higher ratio of Gr 0 toxicity. This is incompatible with the results of Alashkham et al.^(17)^, who reported significantly higher rates (p<0.001) of high-grade proctitis in diabetic prostate patients after pelvic radiotherapy and suggested diabetes as a significant predictor of proctitis after pelvic radiotherapy. Another study by Zelefsky et al.^(9)^ also reported a significant association between late gastrointestinal and urinary toxicities with diabetes in patients with localized prostate cancer treated with 3DCRT, in conformity with our findings. The results of our data analysis revealed that the incidence of proctitis was significantly lower in patients who were not diabetic. A study on the impact of diabetes on radiation toxicity suggested that diabetic status increased the risk of radiation toxicity and reset the onset of symptoms to an earlier time and slowed down its resolution^(17)^. The lower incidence of LGIS in patients with diabetes may be attributed to the scant number of diabetic cases and variable extrinsic factors influencing acute toxicity symptoms if one takes into consideration that previous studies suggested on late toxicities. Radiation dose and technique such as 3DCRT or IMRT may affect radiation toxicity. However, dose was not found to be significant in our study. We were unable to investigate differences between radiation toxicity in insulin or medication using patients with diabetes because all of our patients used oral medications. This issue was mentioned in the study by Kalakota et al.^(8)^ and the authors reported no difference in the incidence of gastrointestinal and urinary toxicity between insulin or oral medications. To the best of our knowledge, no evidence has been presented in terms of the association between LGIS toxicity and age^(18)^; however, we found a significant correlation between age and Gr 0 UGIS toxicity using Pearson’s correlation analysis (p=0.047). A major issue of published literature is the lack of any uniform and reliable method to evaluate and record proctitis, a number of studies did not even record the severity as grades^(19,20,21,22,23,24)^. In this study, we used the RTOG grading system to evaluate the severity of radiation-induced proctitis^(25,26)^ as a reliable and well-recognized method. All our patients were treated with a median pelvic dose of 5040 (range, 4500-6120) cGy, the total dose to tumor or tumor bed was 5040 (range, 4500-6120) cGy in a median 28 (range, 25-32) days; therefore the data were inadequate for evaluating toxicity in patients with diabetes according to higher or lower radiation doses.

One of the limitations of our study is the limited number of patients, which led to inadequate data to enable comment, especially due to the small group with diabetes. The correlations we found and prediction values may be discordant, which can be attributed to the limited number of cases. The second important limitation is the retrospective design, which causes doubts in the uniformity of toxicity data. However, this can be used as a model for pilot studies because this paper is the first to investigate the impact of diabetes on radiation toxicity in women who underwent pelvic +/- paraaortic radiotherapy for gynecologic cancer.

## Conclusion

Our results revealed a significantly lower incidence of LGIS toxicity in nondiabetic patients.

Taking the correlation we found between toxicity and concurrent chemotherapy or paraaortic radiation into consideration, special care should be given to patients with diabetes and risk stratification before radiotherapy. Further prospective studies are recommended to evaluate late toxicity in patients with diabetes, taking the longevity of diabetes history into consideration, and to investigate whether it is controlled using specific laboratory data such as glycated hemoglobin.

## Figures and Tables

**Table 1:**
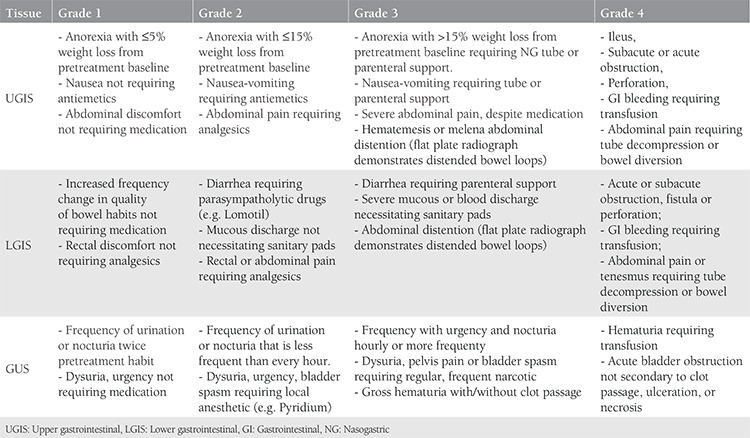
Radiation Oncology Toxicity Grading toxicity grade scoring system

**Table 2:**
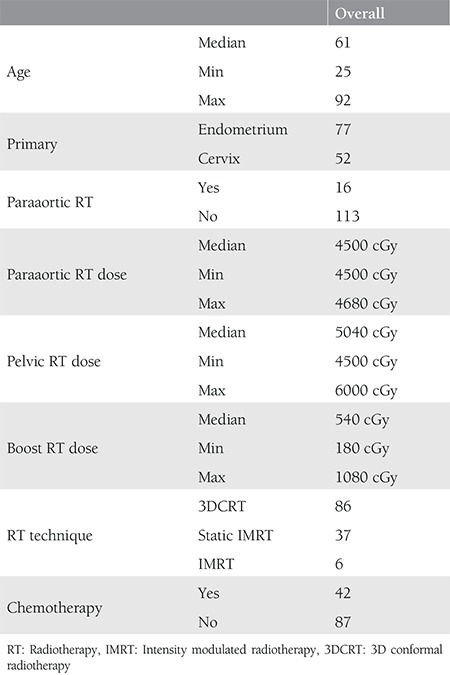
Clinical and demographic characteristics of the patients

**Table 3:**
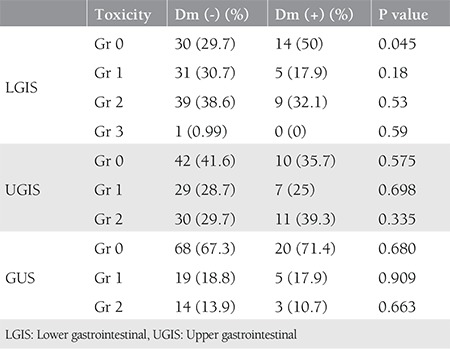
Lower gastrointestinal, upper gastrointestinal and GUS toxicity with diabetes and p values

**Table 4:**
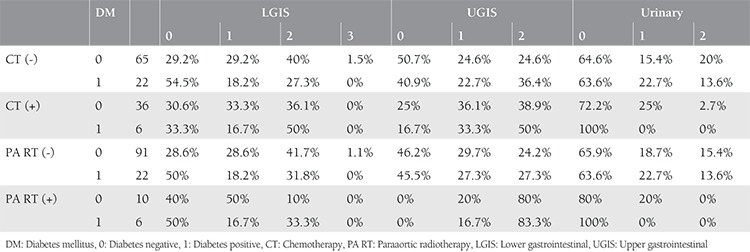
Upper gastrointestinal, lower gastrointestinal and urinary toxicity ratios in diabetic and non-diabetic patients according to chemotherapy and paraaortic irradiation

**Table 5:**
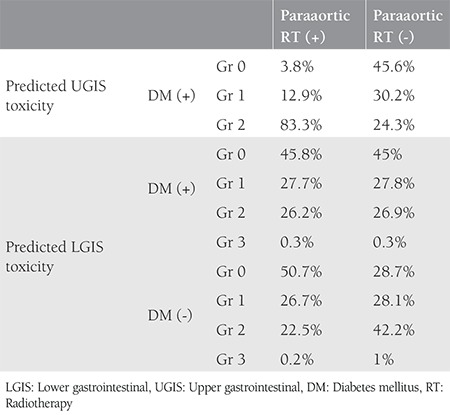
Predicted UGIS and LGIS toxicities with diabetes and paraaortic irradiation
